# Draft genome sequence data of *Haemaphysalis longicornis* Oita strain

**DOI:** 10.1016/j.dib.2023.109352

**Published:** 2023-06-28

**Authors:** Rika Umemiya-Shirafuji, Xuenan Xuan, Kozo Fujisaki, Junya Yamagishi

**Affiliations:** a National Research Center for Protozoan Diseases, Obihiro University of Agriculture and Veterinary Medicine, Obihiro, Hokkaido, 080-8555, Japan; b National Agricultural and Food Research Organization, Kannondai 3-1-5, Ibaraki 305-0856, Japan; c International Collaboration Unit, International Institute for Zoonosis Control, Hokkaido University, Sapporo, Hokkaido, 001-0020, Japan

**Keywords:** Tick, *Haemaphysalis longicornis*, Bisexual race, Laboratory colony, Whole genome sequencing, Nanopore

## Abstract

*Haemaphysalis longicorni*s Neumann, 1901 is one of the most well-known hard ticks because of its medical and veterinary importance. *Haemaphysalis longicorni*s transmit a wide range of pathogens among vertebrates, affecting humans and animals in Asia and Oceania. In Japan, the tick species is a major pest of cattle because it can spread a protozoan parasite *Theileria orientalis*, which causes theileriosis and produces economic losses to the livestock industry (Yokoyama et al. 2012 [1]). Apart from bovine theileriosis, *H. longicornis* is a vector of bovine babesiosis caused by *Babesia ovata*, canine babesiosis caused by *Babesia gibsoni*, and rickettsiosis and viral diseases in humans. Its habitats are mainly Japan, Australia, New Zealand, New Caledonia, the Fiji Islands, Korea, China, and Russia (Oliver et al. 1973 [2]). In the United States, heavy *H. longicornis* infestations on cattle and white-tailed deer were reported in 2019, making it now one of the tick species to be an increasing threat to livestock animals and humans globally.

Ticks reproduce offspring after mating with female and male ticks, however, interestingly, there are two races of *H. longicornis*: bisexual (diploid) and parthenogenetic (triploid) races [2]. Parthenogenetic *H. longicornis* is distributed throughout Japan, while the northern limit of the bisexual race is believed to be Fukushima Prefecture on Honshu Island (Fujita et al. 2013 and Kitaoka et al. 1961 [3,4]). This tick species is also considered to be of great scientific importance, and the parthenogenetic race collected in Okayama prefecture has been reared since 1961, while the bisexual race collected in Oita prefecture has been reared since 2008 under laboratory conditions in Japan (Boldbaatar et al. 2010 and Fujisaki et al. 1976 [5,6]). Namely, the “Okayama strain” and “Oita strain” of *H. longicornis* have been maintained for more than six decades and 15 years, respectively, stably under laboratory conditions. To obtain reference data of bisexual *H. longicornis*, we sequenced unfed females with haploid genomes using Illumina and MinION Q20 kit then obtained a draft genome consisting of 2.48 Gbp. The number of the contig was 98,529 and N50 was 46.5 Kb. Genome information derived from our laboratory colony of bisexual *H. longicornis* ticks would provide fundamental insight into understanding how different reproductive lineages occur within the same species of the tick.

**Specifications Table**

**Table:** 

Subject	Entomology and insect science
Specific subject area	Genomics
Type of data	Table Figure DNA sequence
How the data were acquired	*Haemaphysalis longicornis* bisexual diploid Oita strain genomic DNA was extracted from 50 unfed female ticks. Both Illumina HiSeq X Ten using TruSeq DNA PCR Free and Oxford nanopore FLO_MIN106 flowcells and Q20 Early Access Kit (SQK-Q20EA). The Illumina reads were assembled using AbySS-pe (version 2.1.5). The nanopore reads were assembled using wtdbg2 (version 2.5) then error correction was performed using NextPolish (v1.1.0) supported by the Illumina reads. These contigs were integrated using SAMBA bundled in MaSuRCA (version 4.0.9).
Data format	Raw sequence reads in fastq format de novo assembled sequence in fasta format
Description of data collection	The bisexual *H. longicornis* was collected from Oita Prefecture, Japan in 2008 [5] and maintain at the National Research Center for Protozoan Diseases, Obihiro University of Agriculture and Veterinary Medicine (OUAVM), Obihiro, Japan [5]. Their genomic DNA was extracted from 50 unfed female ticks.
Data source location	Institution: National Research Center for Protozoan Diseases, Obihiro University of Agriculture and Veterinary Medicine, Japan City/Town/Region: Obihiro, Hokkaido Country: Japan
Data accessibility	Repository name: National Center for Biotechnology Information Data identification number: Bioproject ID: PRJNA855600 Biosample ID: SAMN29498733 NCBI GenBank assembly accession: GCA_029849285.1 NCBI GenBank Accession Number of the genome assembly: JANDBB000000000.1
	NCBI SRA Accession Number of the illumiina reads: SRR20011411 NCBI SRA Accession Number of the nanopore reads: SRR20007197 - SRR20007222 Direct URL to data: BioProject: https://www.ncbi.nlm.nih.gov/bioproject/PRJNA855600 BioSample: https://www.ncbi.nlm.nih.gov/biosample/SAMN29498733 Assembly: https://www.ncbi.nlm.nih.gov/assembly/GCA_029849285.1/ Nucleotide: https://www.ncbi.nlm.nih.gov/nuccore/JANDBB000000000 SRA(Illumina): https://www.ncbi.nlm.nih.gov/sra/SRR20011411 SRA (nanopore): from https://www.ncbi.nlm.nih.gov/sra/SRR20007197 to https://www.ncbi.nlm.nih.gov/sra/SRR20007222
Related research article	Not applicable

## Value of the Data

• *Haemaphysalis longicornis* is a vector of pathogens of public health importance; therefore, their genome information can be used to develop countermeasures against the diseases.
• There are two races of *H. longicornis*: bisexual diploid and parthenogenetic triploid. Our data provides genomic information on the former and it should be a basis for understanding how different reproductive lineages occur in the same species of tick.
• In our laboratory, we have maintained bisexual races of *H. longicornis* for over 15 years for studies of tick biology and physiology. Data were obtained from the female ticks, which have been stably reared under laboratory conditions. It can be distributed to the research community as a bioresource. Therefore, genome information and biological resources are available as a set.
• Providing stable laboratory strains together with their genome information will be useful resources to the tick research community. These can be used for further studies to elucidate the molecular basis of reproduction, pathogen vector competency, acaricide development, spatio-temporal distribution, and so on.

## 1 Objective

*Haemaphysalis longicornis* ticks distributed in Asia and Oceania transmit various pathogens to vertebrates, which is an increasing threat to livestock and humans even in the United States [7]. So far, three genome sequences of *H. longicornis* are available to the public. Larvae hatched from a single wild-collected female in China were used for the first genome with 2.55 Gb (https://www.ncbi.nlm.nih.gov/nuccore/JABSTR000000000) [8]. Subsequently, they sequenced individual *H. longicornis* males and females resulting in the draft genome with 2.4-2.8 Gb and 3.6 Gb in size. Another genome with 3.16 Gb has been sequenced using unfed females after six generations of a pair of wild-collected male and female ticks in China (https://www.ncbi.nlm.nih.gov/nuccore/2200760349) [9]. Eggs from New Zealand-collected females have been sequenced and a genome with 7.36 Gb has been obtained (https://www.ncbi.nlm.nih.gov/nuccore/VFIB00000000) [10]. Interestingly, the size of the genome of *H. longicornis* ticks from each country is inconsistent as described above. Their bisexual or parthenogenetic reproduction and related ploidy might explain the diversity but the detail remains unclear. Besides, none of them have been obtained from laboratory colonies sustained for a long period. Therefore, we attempted to provide draft genome of the bisexual *H. longicornis* (Oita strain) which has been maintained for over 15 years as reference data.

## 2 Data Description

So far, three genome sequences are available in public [8], [9], [10]. Larvae hatched from a single wild collected female in China were used for the first genome with 2.55 Gb [8]. In addition, they sequenced individual *H. longicornis* male and female resulted in draft genome with 2.4-2.8 Gb and 3.6 Gb in size. Another genome with 3.16 Gb has been sequenced using unfed female after six generations of wild collected single male and female pair in China [9]. Eggs from New Zealand-collected females has been sequenced and a genome with 7.36 Gb has been obtained [10]. Interestingly, the size of genome is inconsistent as shown above. Their mode of reproduction, gamogenesis or parthenogenesis, and related ploidy might explain the diversity but the detail is not clear. Besides, none of them have been obtained from laboratory colonies sustained for a long period of time.

**Fig. 1:**
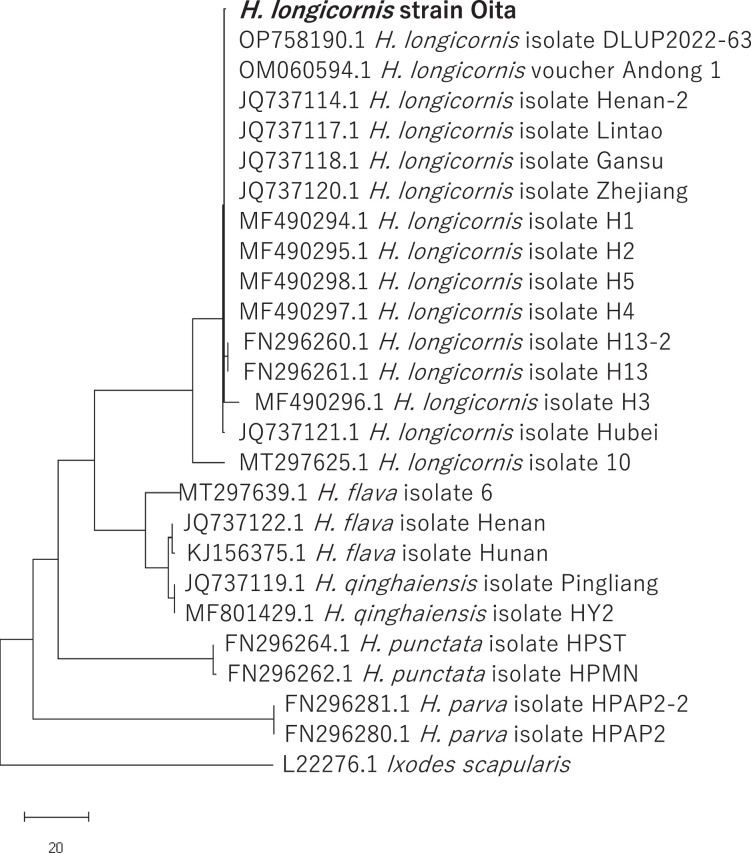
Phylogenetic tree based on ITS-1 sequences. The ITS-1 sequence in the *H. longicornis* Oita strain draft genome were aligned with 24 representative ITS-1 sequences of *Haemaphysalis* spp. together with *Ixodes scapularis* ITS-1.

Here, we provide genomic information for the Oita laboratory strain. Genomic DNA was purified from fifty unfed female ticks. The DNA was sequenced using 12 MinION flowcells using Q20 library preparation kit and Illumina HiSeq X ten (Table 1). The MinION reads were assembled by wtdbg2 [11] then polished by NextPolish [12] using the Illumina reads. The Illumina reads were assembled by AbySS-pe [13]. The resulted contigs were integrated by SAMBA. As a result, we obtained a draft genome consisted of 2.48 Gbp and 98,529 contigs (Table 2). Its N50 was 46.5 Kb. Average read depth were 51.8 for the Illumina reads and 19.1 for the nanopore reads, respectively. Its completeness was examined by BUSCO [14] and 75.3%, 11.6%, and 13.1% of complete, fragmented, and missing genes were detected, respectively (Table 2). It was confirmed that the genome belonged to *H. longicornis* for sure by phylogenetic analysis using Internal transcribed spacer 1 (ITS-1) (Fig. 1). The assembled genome is available at the National Center for Biotechnology Information (NCBI) through the accession JANDBB000000000.1 (https://www.ncbi.nlm.nih.gov/nuccore/JANDBB000000000.1). The raw read data for Illumina and MinION is available through SRR20011411 (https://trace.ncbi.nlm.nih.gov/Traces/sra/?run=SRR20011411) and SRR20007197 - SRR20007222, respectively (https://trace.ncbi.nlm.nih.gov/Traces/sra/?run=SRR20007197 - https://trace.ncbi.nlm.nih.gov/Traces/sra/?run=SRR2000222). The BioProjectID is PRJNA855600 and BioSample accession is SAMN29498733.

**Table 1:** Statistics of the sequenced reads.

**MinION:**	
Total number of MinION flowcells	12
Total reads	21,972,339
Total bp	62,048,804,095
Average bp	2,824
Read depth	19.1
**Illumina:**	
Total reads	1,743,927,178
Read depth	51.8
Total bp	263,333,003,878

**Table 2:** Feture of the assebbled genome for H. longicornis Oita strain.

Total length	2,476,780,723 bp
Number of contigs	98,529
contig N50	46,512 bp
Largest contig	641,034 bp
GC content	47.4%
Number of BUSCO Complete and single-copy (% of total)	771 (76.1%)
Number of BUSCO Complete and duplicated (% of total)	25 (2.7%)
Number of BUSCO Fragmented (% of total)	133 (13.1%)
Number of BUSCO Missing (% of total)	84 (8.3%)

## 3 Experimental Design, Materials and Methods

### 3.1 Tick and isolation of genomic DNA

The bisexual *H. longicornis* was collected from Oita Prefecture, Japan in 2008 [5]. The ticks have been maintained by blood-feeding on Japanese white rabbits (female, specific-pathogen-free; Japan SLC, Shizuoka, Japan) at the National Research Center for Protozoan Diseases, Obihiro University of Agriculture and Veterinary Medicine (OUAVM), Obihiro, Japan. Rabbits were reared in a temperature- and humidity-regulated room under controlled lighting, water, and commercial regular chow (CLEA Japan, Tokyo, Japan).

Fifty unfed female ticks were used for the extraction of genomic DNA. Female ticks were immersed in 10 ml of 0.5% sodium hypochlorite in PBS to disinfect the surface of ticks. After 30 min, ticks were rinsed with PBS for 5 min 3 times. Ticks were homogenized with liquid nitrogen in an autoclaved mortar and suspended in 5 ml of DNA extraction buffer. Genomic DNA was extracted using the suspension by a standard procedure [15]. The genomic DNA sample treated with RNase A to avoid contamination was kept at -30°C until use.

### 3.2 DNA sequencing

Oxford nanopore Q20 system (SQK-Q20EA) with increased accuracy is available. We sequenced the DNA using they system together with 12 flowcells (FLO_MIN106). Following base calling was performed using guppy version 5.0.16+ with argument -c res_dna_r9.4.1_e8.1_sup_v033.cfg. Sequencing with Illumina platform was also performed using one lane of the Illumina HiSeq X ten with TruSeq DNA PCR Free library preparation kit.

### 3.3 Assembly and analysis

Reads obtained from the MinION system were trimmed by porechop 0.2.3_seqan2.1.1 with default argument [16]. The trimmed reads were assembled by wtdbg2 2.5 followed by wtpoa-cns [11] with arguments -L 1000 -g 2.5g -p 21 -k 0 -AS 4 -K 0.05 -s 0.05 -e 1. Error correction was performed using nextPolish [12] with the default arguments. Reads obtained from the Illumina platform were trimmed by trimmomatic-0.36 [17] with arguments PE LEADING:20 TRAILING:20 SLIDINGWINDOW:4:15 MINLEN:36. The trimmed reads were assembled by abyss-pe 2.1.5_1 with arguments k=96 j=256 B=50G H=4 kc=2 v=-v. Resulted contigs were integrated by SAMBA which bundled with MaSuRCA-4.0.9 [18] with arguments -t 64 -d asm -m 1500 -o 3000. Sequence depth for the Illumina and nanopore reads was calculated by BamDeal (https://github.com/BGI-shenzhen/BamDeal) using alignments obtained by bowtie2 version 2.3.4.1 [19] and minimap2 version 2.24 [20], respectively. Validation of completeness was performed by BUSCO v5.3.2_cv1 [14] with arguments -m genome -l arthropoda_odb10. Internal transcribed spacer 1 sequences were identified using blastn homology search for known *H. longicornis* isolate He13 ITS-1 sequence (MF490336.1). The most identical sequence was selected and aligned with 24 representative ITS-1 sequences of *Haemaphysalis* spp. together with *Ixodes scapularis* ITS-1 (L22276.1) as an out group using muscle [21]. Gaps were eliminated and the phylogenetic tree was described using neighbor-joining method [22].

## Ethics Statements

The experimental design and management of rabbits used for tick infestation were approved by the Experimental Animal Committee of Obihiro University of Agriculture and Veterinary Medicine (Animal experiment approval number: 19-74). This work does not contain any studies with human subjects.

## CRediT authorship contribution statement

**Rika Umemiya-Shirafuji:** Writing – original draft. **Xuenan Xuan:** Conceptualization, Writing – review & editing. **Kozo Fujisaki:** Writing – original draft. **Junya Yamagishi:** Conceptualization, Supervision.

## Declaration of Competing Interest

The authors declare that they have no known competing financial interests or personal relationships that could have appeared to influence the work reported in this paper.

## Data Availability

Draft genome sequence data of Haemaphysalis longicornis Oita strain (Original data) (genbank).
